# Dosimetry of the brain and hypothalamus predicting acute lymphopenia and the survival of glioma patients with postoperative radiotherapy

**DOI:** 10.1002/cam4.2159

**Published:** 2019-04-14

**Authors:** Lu‐Lu Ye, Xing‐Wen Fan, Chao‐Su Hu, Xia‐Yun He, Xiao‐Shen Wang, Chun‐Ying Shen, Ting‐Ting Xu, Hong‐Mei Ying

**Affiliations:** ^1^ Department of Radiation Oncology Fudan University Shanghai Cancer Center Shanghai P.R China; ^2^ Department of Oncology Shanghai Medical College, Fudan University Shanghai P.R China

**Keywords:** dosimetric predictor, glioma, hypothalamus, lymphocyte, whole brain

## Abstract

**Background:**

The aim of this study was to investigate dosimetric factors for predicting acute lymphopenia and the survival of glioma patients with postoperative intensity‐modulated radiotherapy (IMRT).

**Methods:**

A total of 148 glioma patients were reviewed. Acute lymphopenia was defined as a peripheral lymphocyte count (PLC) lower than 1.0 × 10^9^/L during radiotherapy with a normal level at pretreatment. PLCs with the corresponding dates and dose volume histogram parameters were collected. Univariate and multivariate Cox regression analyses were constructed to assess the significance of risk factors associated with lymphopenia and overall survival (OS).

**Results:**

Sixty‐nine (46.6%) patients developed lymphopenia during radiotherapy. Multivariate analyses revealed that the risk increased with the maximal dose of the hypothalamus (HT Dmax) ≥56 Gy (58.9% vs 28.5%, *P* = 0.002), minimal dose of the whole brain (WB Dmin) ≥2 Gy (54.3% vs 33.9%, *P* = 0.006), or mean dose of the WB (WB Dmean) ≥34 Gy (56.0% vs 37.0%, *P* = 0.022). Patients with older age, high‐grade glioma, development of lymphopenia, high HT Dmax, WB Dmin, and WB Dmean had significantly inferior OS in the multivariate analyses.

**Conclusions:**

HT Dmax, WB Dmin, and WB Dmean are promising indicators of lymphopenia and the survival of glioma patients undergoing postoperative IMRT. The necessity and feasibility of dosimetric constraints for HT and WB is warranted with further investigation.

## 1 INTRODUCTION

The pivotal role of immune surveillance against neoplasms is well known.^1^, ^2^ However, immunosuppression elicited by therapy is frequently observed in patients with various malignancies, such as those of the pancreas, breast, uterine cervix, and brain.^3^, ^4^, ^5^, ^6^, ^7^ This iatrogenic lymphopenia was not taken seriously until subsequent evidences indicated that patients with lowered peripheral lymphocyte counts (PLCs) suffered early death from tumor progression.^8^ Recent studies have brought attention to the treatment‐related lymphopenia observed in glioma patients.^9^, ^10^, ^11^, ^12^, ^13^, ^14^, ^15^, ^16^ As the standard of care for gliomas, radiotherapy (RT), steroids,^4^ and temozolomide (TMZ)^17^ are well‐documented lymphotoxins. This comprehensive therapy modality causes 40% of high‐grade glioma (HGG) patients to develop severe lymphopenia (grade III‐IV toxicity) within 2 months after course initiation.^11^ The state of immunosuppression is persistent^11^, ^13^, ^15^ and detrimentally affects patients with increased susceptibility to opportunistic infections,^9^ additional risk of secondary central nervous system (CNS) tumors,^18^ and deteriorated survival time.^11^

RT‐related lymphopenia is substantial and results primarily in deleterious effects on CD4^+^ T cells^11^, ^12^, ^13^, ^15^, ^16^ and natural killer (NK) cells.^12^, ^15^, ^16^ The direct destruction of lymphocytes in the radiation field of the brain has been suggested as a possible mechanism. Lymphocytes are extremely radiosensitive to an exposure dose of 0.5 Gray (Gy), with a lethality rate of 10%.^19^, ^20^ Preclinical studies have verified the critical role that the irradiation of circulating blood plays in the etiology of lymphodepletion.^21^, ^22^ The PLC is reduced as the number of fractions increases in patients with brain irradiation.^25^ Yovino et al^26^ developed a mathematical model of an RT plan for HGG patients. A single fraction of 2 Gy delivers a lymphotoxic dose of 0.5 Gy^20^ to 5% of circulating lymphocytes, whereas a typical course of 30 fractions affects 99% of lymphocytes exposed to at least 0.5 Gy. In addition, Huang et al^14^ states that the dose to brain is an independent indicator of lymphopenia in HGG patients.

Is there a specific tissue that, upon being damaged by irradiation, contributes to immune dysfunction? The hypothalamus (HT) is well known to be a crucial immunoregulatory center.^27^, ^28^ In a series of preclinical studies on the hypothalamic regulation of peripheral immune functions, lesions of discrete areas evoke immune changes.^29^, ^30^ Typically, clinical data have defined the relatively radiosensitive response of the HT with an increased risk of neuroendocrine sequelae after irradiation for brain tumors, where the HT is not deliberately spared within the irradiated volume.^35^, ^36^ This serves as a rational for directing attention to potential dose toxicity to the HT that may be correlated with immunosuppression. Given the undefined mechanisms and scarce data, this retrospective study was aimed to preliminarily investigate the possible dosimetric predictors of acute lymphopenia (AL) and survival outcomes.

## 2 METHODS AND MATERIALS

### 2.1 Patient population

This retrospective study was approved by the Institutional Review Board and performed in accordance with the principles of the Declaration of Helsinki and its amendments. Due to retrospective nature of the study, which only the clinical and dosimetric data bases were studied and very small risks to the patients involved, we requested and were granted a waiver of written informed consent from the institutional review board.

The medical records of patients with newly diagnosed World Health Organization (WHO) grades II to IV glioma undergoing postoperative intensity‐modulated radiotherapy (IMRT) at the Fudan University Shanghai Cancer Center between April 2007 and December 2016 were reviewed. The exclusion criteria were (a) history of irradiation to the head and neck; (b) age below 16 years; (c) Karnofsky performance score <70; (d) open or stereotactic biopsy only; (e) previously confirmed hematological diseases or a PLC below 1.0 × 10^9^/L at diagnosis; (f) incomplete blood test data before and during RT; (g) incomplete prescribed RT; (h) follow‐up for less than 1 year; and (i) inaccessible RT dosimetric data. The pretreatment workup included a comprehensive physical examination; postoperative evaluation with magnetic resonance imaging (MRI) scans of the brain; computed tomography (CT) scan of the chest; abdominal ultrasound; and blood samples for hematological, biochemical, and serological estimations.

### 2.2 Radiotherapy and chemotherapy

Patients were immobilized with a thermoplastic head mask that ensured reproducible positioning. A CT scan with intravenous contrast was performed on a CT simulator (Brilliance CT Big Bore, Philips Healthcare, Cleveland, OH). The CT images were then electronically transferred to the treatment planning system (TPS) and fused to images obtained via contrast‐enhanced T1 and T2 fluid‐attenuated inversion recovery (FLAIR) sequences from postoperative MRI acquired on a magnetic resonance scanner (GE Healthcare, Salem, CT).

The RT course was given in the form of IMRT with 6 megavoltage photons. The treatment was scheduled consecutive 5 d/wk. Gross tumor volume (GTV) was the area of surgical cavity and residual enhancing tumor. Anatomically constrained expansion of the GTV was generated as the clinical target volume (CTV). Another 0.3‐0.5 cm expansion was added to the CTV to allow for error setup and movement during the RT course, to establish the planning target volumes (PTV).

In low grade glioma (LGG), the GTV was defined as the area of surgical cavity and residual contrast enhancement on contrast‐enhance T1 and T2‐FLAIR sequences. A margin, usually 1‐2 cm, was added to the GTV to define the CTV. The total dosage was 54 Gy in a daily fraction of 1.8‐2.0 Gy. An appropriate boost not exceeding 60 Gy was considered in cases of incomplete resection. With regard to HGG, the GTV consisted of surgical cavity and any residual contrast enhancement on contrast‐enhance T1 but ignoring any edema. The PTV1 represented 0.3‐0.5 cm expansion of the CTV, which was typically 2 cm expansion of the GTV, and the PTV2 was employed as the GTV together with 0.5 cm margin around it. A total dosage of 60 Gy in a daily fraction of 2.0 Gy was adopted in two consecutive courses. The first course with 50 Gy in 25 fractions was delivered to the PTV1, and the second course of 10 Gy in 5 fractions was given to the PTV2.

The dose constraints for organs at risk (OAR) were as follows: the maximum doses to the brainstem, optic nerves and optic chiasm were ≤54 Gy; that to the spinal cord was ≤45 Gy; that to the lens was ≤6 Gy; and the mean dose to the eyeballs was ≤35 Gy.

Concomitant TMZ at a daily dose of 75 mg/m^2 ^was given from the initiation of RT. After a 4‐week break, adjuvant TMZ at 150‐200 mg/m^2^ for 5 consecutive days every 28 days was given for up to 6 cycles.

### 2.3 Lymphocyte evaluation

Routine blood tests that were assessed within 14 days before initiation of RT were recorded as baseline data. PLCs that were determined every week during RT were recorded with the corresponding dates. The PLC was measured with an automated hematology analyzer Sysmex XT‐4000i (Sysmex, Kobe, Japan).

### 2.4 Dose volume histogram parameters

The delineations of the HT, hippocampus (HC) and whole brain (WB) were performed by two experienced radiation therapists who were blinded to the patients’ medical information. When significant disagreement occurred, a third radiation therapist was needed to minimize the deviation.

The HT was contoured from the hypothalamic sulcus at the level of the anterior commissure downwards to the end of the third ventricle. An additional 3‐mm margin was expanded to encompass the entire third ventricle.^37^ The HC was countered according to RTOG 0933.^38^ Briefly, the gray matter within the curve of the temporal horn at the level of the temporal horn and that bound upwards by cerebrospinal fluid in the lateral ventricle and the ambient cistern were contoured. However, below the level of the temporal horn, the HC was indistinct from the amygdala in terms of the boundary, so the delineation was extrapolated from superior slices. The WB was routinely contoured from the top of the skull to the foramen magnum.

Dose distributions were calculated with the collapse cone convolution algorithm in Pinnacle TPS (version 9.0, Philips Radiation Oncology Systems, Fitchburg, WI). The PTV of the tumor bed and dose volume parameters of HT, HC, and WB were calculated as follows: minimal dose (Dmin), maximal dose (Dmax), and mean dose (Dmean).

### 2.5 Follow‐up and endpoints

After the completion of treatment, patients received regular examinations at outpatient clinics at 3‐month intervals during the first 2 years, every 6‐9 months during their 3rd to 5th years and annually thereafter.

The primary endpoint was AL, and the secondary endpoint was overall survival (OS). AL was defined as a PLC below 1.0 × 10^9^/L during RT, and the toxicity grade was guided by Common Terminology Criteria for Adverse Events version 5.0 (CTCAE v5.0). The time of AL onset was the interval between the initiation of RT and the first abnormal record of PLC. OS was defined as the time interval between the initial therapy and the date of death. For patients who were still alive, the latest follow‐up date was recorded.

### 2.6 Statistical analysis

The Statistical Package for the Social Sciences version 22.0 (IBM, Armonk, NY) was used for the data analysis. The incidence of AL and the survival curves for OS were obtained utilizing the Kaplan‐Meier method with the log‐rank test. Numeric data were averaged and expressed as the mean ± standard deviation. The chi‐square test or Fischer's exact test was performed to determine the correlation of categorical variables with AL, while the Mann‐Whitney *U* test was carried out to assess the relationship between numerical variables and AL. The Pearson correlation coefficient was used to assess correlations between variables. Dosimetric parameters were dichotomized by median value as the cutoff points. Univariate and multivariate Cox proportional hazards regression analyses were carried out to assess the significance of variables associated with clinical outcomes. All clinicopathological variables identified in the univariate analysis were entered into the multivariate analysis. Log‐minus‐log plots were used to evaluate the proportional hazard assumption. Any result with a two‐sided *P* value <0.05 was considered statistically significant.

## 3 RESULTS

### 3.1 Basic characteristics

**Figure 1:**
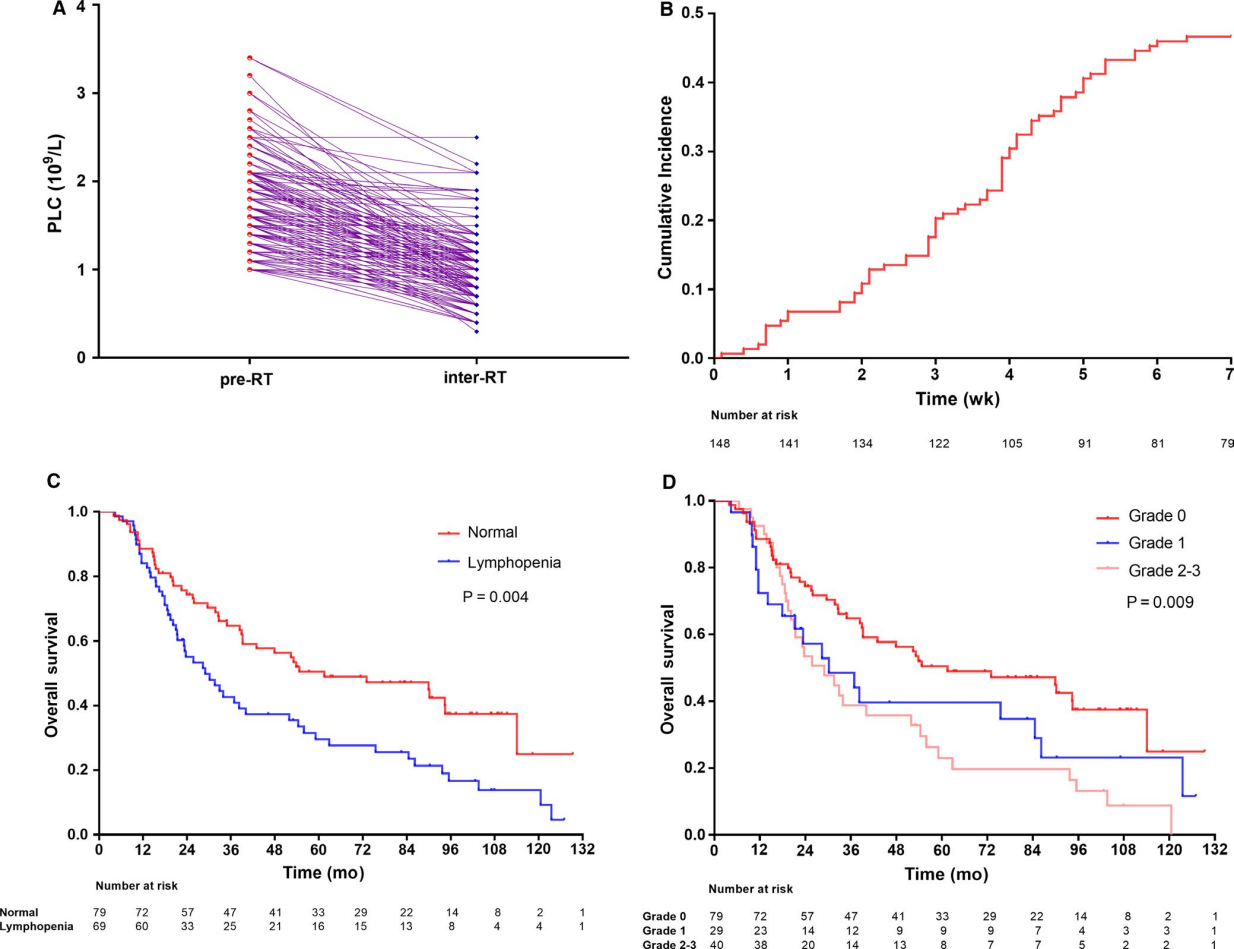
Occurrence of acute lymphopenia during radiotherapy in glioma patients: comparison of the preradiotherapy and interradiotherapy PLCs (A); Kaplan‐Meier curves of acute lymphopenia (B), overall survival according to PLC (C) and toxicity grade of lymphopenia (D). Log‐rank test, *P* < 0.05. Abbreviations: PLC, peripheral lymphocyte count; RT, radiotherapy

Of the 148 patients eligible for this study, 50 (33.8%) patients had WHO‐grade II glioma, 33 (22.3%) had WHO‐grade III glioma, and 65 (43.9%) had WHO‐grade IV glioma. Besides prescribed radiation therapy, 91 (61.5%) patients received TMZ, no one was applied steroids during radiotherapy. Throughout the period of RT, 69 (46.6%) patients developed AL (Figure 1A,B). The median clinical latency for AL was 5.0 weeks (range: 0.1‐6.4 weeks). As depicted in Supplementary Table S1, there were nonsignificant differences in the distributions of basic clinicopathological characteristics between the non‐AL and AL groups, with the exception of concomitant TMZ and the DVH parameters of HT, HC, and WB.

On the whole, during a median follow‐up time of 32.8 months (range: 4.0‐129.3 months), a total of 97 (65.5%) patients died. The 5‐year OS rates were 65.2% for LGG and 28.4% for HGG (Supplementary Figure S2A). The AL groups suffered significantly worse OS than the non‐AL group (median survival: 29.0 vs 61.5 months, 5‐year OS: 29.6% vs 50.5%, *P* = 0.004; Figure 1C). Moreover, the survival trend with the increasing toxicity grade of lymphopenia was dismal (*P* = 0.009; Figure 1D).

### 3.2 Acute lymphopenia

**Figure 2:**
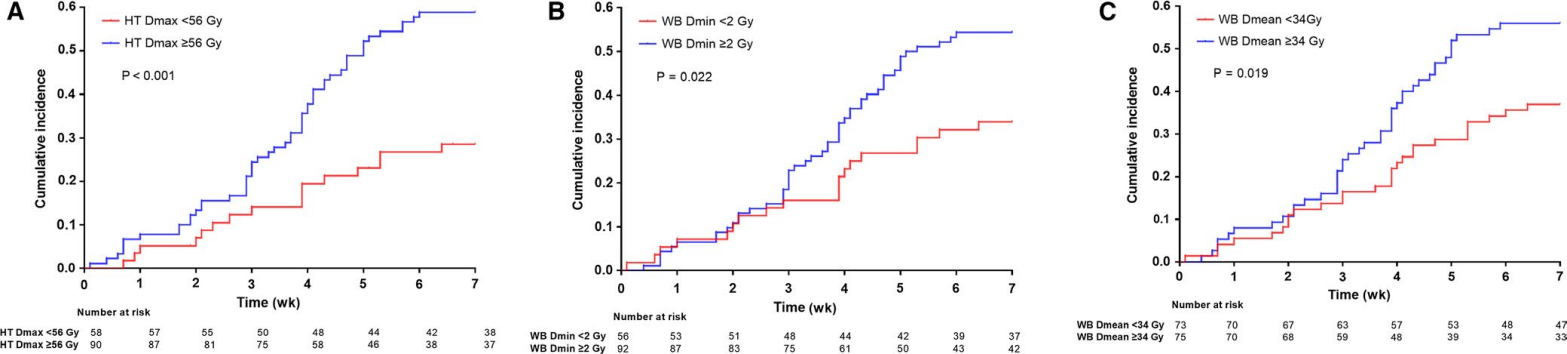
Kaplan‐Meier curves of acute lymphopenia during radiotherapy in glioma patients according to HT Dmax (A), WB Dmin (B) and WB Dmean (C). Log‐rank test, *P* < 0.05. Dmax, maximal dose; Dmean, mean dose; Dmin, minimal dose; Gy, gray; HT, hypothalamus; WB, whole brain

Univariate Cox regression analysis (Table 1) revealed that concomitant TMZ, HT Dmax ≥56 Gy, WB Dmin ≥2 Gy, and WB Dmean ≥34 Gy were significant risk factors for the development of AL. The dosimetric parameters related to HC failed to reach significance. The AL rates were 58.9% vs 28.5% for high vs low HT Dmax (*P* < 0.001; Figure 2A); 54.3% vs 33.9% for high vs low WB Dmin (*P* = 0.022; Figure 2B), and 56.0% vs 37.0% for high vs low WB Dmean (*P* = 0.019; Figure 2C).

**Table 1:** Cox regression analysis with respect to the potential factors of lymphopenia during postoperative radiotherapy

Variables	Unadjusted analysis		Adjusted analysis^a^	
	HR (95% CI)^b^	P value	HR (95% CI)^b^	*P* value
Age (≥60 y)	0.749 (0.383‐1.465)	0.399		
Sex (female)	1.589 (0.989‐2.555)	0.056		
Histological grade (HGG)	1.690 (0.994‐2.873)	0.053		
PTV (≥409 cm^3^)	1.141 (0.711‐1.830)	0.585		
Duration from surgery to radiation (≥6.0 wk)	0.811 (0.506‐1.301)	0.386		
Concomitant temozolomide (yes)	2.224 (1.285‐3.848)	**0.004**		
Hypothalamus DVH (Gy)				
Dmin (≥35)	1.317 (0.818‐2.121)	0.258		
Dmax (≥56)	2.659 (1.518‐4.658)	**0.001**	2.474 (1.384‐4.422)	**0.002** c
Dmean (≥50)	1.365 (0.846‐2.203)	0.203		
Hippocampus DVH (Gy)				
Dmin (≥17)	1.180 (0.735‐1.895)	0.493		
Dmax (≥60)	1.450 (0.900‐2.335)	0.127		
Dmean (≥41)	1.324 (0.820‐2.136)	0.251		
Whole Brain DVH (Gy)				
Dmin (≥2)	1.830 (1.078‐3.106)	**0.025**	2.294 (1.265‐4.160)	**0.006** c
Dmax (≥63)	1.464 (0.882‐2.431)	0.140		
Dmean (≥34)	1.765 (1.087‐2.864)	**0.022**	2.065 (1.109‐3.843)	**0.022** c

Abbreviations: CI, confidence interval; cm^3^, cubic centimetre; Dmax, maximal dose; Dmean, mean dose; Dmin, minimal dose; DVH, dose‐volume histogram; Gy, gray; HGG, high grade glioma; HR, hazard ratio; PTV, planning target volume.

a Adjusted for age, sex, histological grade, PTV, duration from surgery to radiation and concomitant temozolomide.

b Cox proportional hazards model. Bolding shows *P* value <0.05.

c Due to collinearity, these potential variables were entered separately into different multivariate regression models.

Correlations (Supplementary Figure S1) assessed by Pearson correlation coefficients revealed positive correlations among the following three variables: WB Dmin with WB Dmean (r = 0.806, *P* < 0.001), WB Dmin with HT Dmax (r = 0.632, *P* < 0.001), and WB Dmean with HT Dmax (r = 0.799, *P* < 0.001). Because collinearity would lead to highly unstable estimated regression coefficients, these three dosimetric variables were entered separately into different multivariate regression models (Table 2).

**Table 2:** Univariate Cox regression analysis of potential factors affecting the overall survival of glioma patients

Variables	Univariate analysis	
	HR (95% CI)^a^	*P* value
Age (≥60 y)	2.616 (1.631‐4.197)	**<0.001**
Sex (female)	0.739 (0.479‐1.138)	0.169
Histological grade (HGG)	2.964 (1.836‐4.786)	**<0.001**
PTV (≥409 cm^3^)	1.159 (0.775‐1.732)	0.472
Duration from surgery to radiation (≥6.0 wk)	0.733 (0.492‐1.093)	0.128
Concomitant temozolomide (yes)	1.614 (1.057‐2.464)	**0.027**
Lymphopenia (<1.0 × 10^9^/L)	1.735 (1.167‐2.579)	**0.006**
Hypothalamus Dmax (≥56 Gy)	1.653 (1.084‐2.521)	**0.019**
Whole brain Dmin (≥2 Gy)	2.041 (1.305‐3.193)	**0.002**
Whole brain Dmean (≥34 Gy)	1.750 (1.174‐2.608)	**0.006**

Abbreviations: CI, confidence interval; Dmax, maximal dose; Dmean, mean dose; Dmin, minimal dose; Gy, gray; HGG, high grade glioma; HR, hazard ratio; PTV, planning target volume.

a Cox proportional hazards model. Bolding shows *P* value <0.05.

After adjusting for potential variables, high HT Dmax (HR: 2.474, 95% CI: 1.384‐4.422, *P* = 0.002), WB Dmin (HR: 2.294, 95% CI: 1.265‐4.160, *P* = 0.006) and WB Dmean (HR: 2.065, 95% CI: 1.109‐3.843, *P* = 0.022) were still statistically significant risk factors for AL (Table 1).

### 3.3 Survival analysis

**Figure 3:**
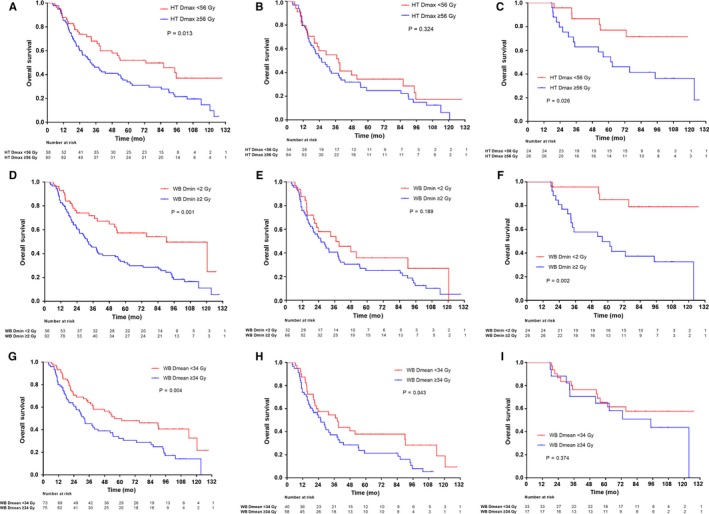
Kaplan‐Meier curves of overall survival according to HT Dmax, WB Dmin and WB Dmean in the whole entity (A, D, G), HGG group (B, E, H) and LGG group (C, F, I). Log‐rank test, *P* < 0.05. Dmax, maximal dose; Dmean, mean dose; Dmin, minimal dose; Gy, gray; HGG, high‐grade glioma; HT, hypothalamus; LGG, low‐grade glioma; WB, whole brain

Patients suffered worse OS with high HT Dmax (median survival: 31.8 vs 71.0 months, 5‐year: 33.9% vs 52.0%, *P* = 0.013; Figure 3A), WB Dmin (median survival: 32.6 vs 90.2 months, 5‐year: 32.3% vs 57.3%, *P* = 0.001; Figure 3D) and WB Dmean (median survival: 31.8 vs 55.9 months, 5‐year: 32.2% vs 49.9%, *P* = 0.004; Figure 3G). Because the critical role of the WHO grading scheme dominates the patient's prognosis, subgroup analyses were carried out. The results showed that HT Dmax and WB Dmin preferentially affected the survival of LGG groups (*P* = 0.026 and *P* = 0.002, respectively; Figure 3), whereas WB Dmean was detrimental to the HGG groups to a greater extent (*P* = 0.043; Figure 3). Additionally, the OS of LGG patients who received nonuniform radiation dosages showed an insignificant difference (*P* = 0.563; Supplementary Figure S2B).

Univariate Cox regression analysis (Table 3) suggested that older age, HGG, concomitant TMZ, lymphopenia, high HT Dmax, WB Dmin, and WB Dmean were significantly associated with survival. Similarly, three multivariate models were established separately for these dosimetric variables. Older age, HGG, lymphopenia, high HT Dmax (HR: 1.598, 95% CI: 1.020‐2.503, *P* = 0.041), WB Dmin (HR: 1.901, 95% CI: 1.139‐3.173, *P* = 0.014), and WB Dmean (HR: 1.914, 95% CI: 1.151‐3.181, *P* = 0.012) were confirmed to be independent prognostic factors for poor OS (Table 3).

**Table 3:** Multivariate Cox regression analysis of potential factors affecting the overall survival of glioma patients

Variables	Multivariate analysis^a^	
	HR (95% CI)^b^	*P* value
Hypothalamus Dmax model^c^		
Age (≥60 y)	2.439 (1.466‐4.057)	**0.001**
Histological grade (HGG)	3.087 (1.739‐5.482)	**<0.001**
Concomitant temozolomide (yes)	0.814 (0.486‐1.366)	0.437
Lymphopenia (<1.0 × 10^9^/L)	1.936 (1.343‐3.019)	**0.004**
Hypothalamus Dmax (≥56 Gy)	1.598 (1.020‐2.503)	**0.041**
Whole brain Dmin model^c^		
Age (≥60 y)	2.388 (1.444‐3.948)	**0.001**
Histological grade (HGG)	2.986 (1.658‐5.378)	**<0.001**
Concomitant temozolomide (yes)	0.827 (0.488‐1.401)	0.479
Lymphopenia (<1.0 × 10^9^/L)	1.927 (1.234‐3.008)	**0.004**
Whole brain Dmin (≥2 Gy)	1.901 (1.139‐3.173)	**0.014**
Whole brain Dmean model^c^		
Age (≥60 y)	2.362 (1.427‐3.909)	**0.001**
Histological grade (HGG)	2.946 (1.645‐5.278)	**<0.001**
Concomitant temozolomide (yes)	0.873 (0.514‐1.480)	0.613
Lymphopenia (<1.0 × 10^9^/L)	1.758 (1.110‐2.786)	**0.016**
Whole brain Dmean (≥34 Gy)	1.914 (1.151‐3.181)	**0.012**

Abbreviations: CI, confidence interval; Dmax, maximal dose; Dmean, mean dose; Dmin, minimal dose; Gy, gray; HGG, high grade glioma; HR, hazard ratio; PTV, planning target volume.

a The following variables were entered into each model: age, sex, histological grade, PTV, duration from surgery to radiation, concomitant temozolomide and lymphopenia.

b Cox proportional hazards model. Bolding shows *P* value <0.05.

c Due to collinearity, these three dosimetric variables were entered separately into different multivariate regression models.

## 4 DISCUSSION

Our study was the first to suggest that the irradiation doses to the HT and WB could predict not only lymphopenia but also the survival of patients with glioma receiving postoperative RT. The current data demonstrate that the incidence of AL is 46.6% during the RT course and significantly decreases patient survival, which is in accordance with previous findings.^11^ We identified HT Dmax, WB Dmin, and WB Dmean as significant dosimetric parameters that are associated with the risk of lymphopenia. Notably, a higher dose to the HT or WB is a passive prognostic indicator for survival.

Lymphocytes are known to be particularly radiosensitive,^19^, ^20^ where DNA fragmentation occurs at low radiation doses.^39^ There is broad concern that traditional fractionated radiation can hinder immunity^9^, ^10^, ^11^ in part due to the exposure of lymphocytes to radiation within RT fields. The PLC is incrementally reduced by 5%‐6% with each additional fraction in patients with brain irradiation.^25^ This strongly implicated that radiation‐induced lymphopenia is correlated with the dose received by circulating blood. Similar to our results, the findings by Tang et al^40^ suggest that systemic lymphodepletion is correlated more with lower dose ranges. Given these findings, a minimal dose to the brain is believed to be vital in lymphodepletion.

A question of particular interest in this study was whether irradiation to specific tissues of the CNS elicited immune dysfunction, and the results support this notion. Moreover, murine models receiving brain irradiation exhibit parallel changes in their immune organs and lymphatic tissues outside of the RT field.^23^, ^24^ Direct injury to circulating lymphocytes is not a sufficient explanation of this phenomenon. A previous study revealed profound lymphopenia with a sharp reduction predominantly in circulating CD4^+^ T cells after focal irradiation in patients with breast cancer and seminoma testis.^7^ Similar alterative patterns in circulating lymphocytes have been observed in glioma patients treated with RT.^11^, ^12^, ^13^, ^15^ In addition, a striking drop in the NK cell population has also been detected in this setting,^12^, ^15^, ^16^ of which the lymphocytic phenotype is consistent with baseline after irradiation to other sites.^7^ On the other hand, preclinical data have indicated that the hypothalamic effects on immunomodulation affect NK cells predominantly. Excision of hypothalamic nuclei^29^, ^30^ leads to persistent reductions in the cytotoxic activity of NK cells and the population of lymphocytes in both the peripheral blood and spleen. As such, these reductions may be a mechanistic extrapolation that NK cell‐specific lymphopenia may be attributable to radiation damage to the HT, which has similarly been suggested in direct ablation studies. The HC, which is similar to the HT in terms of both location and a high correlation with the dose to the brain (Supplementary Figure S1), is insignificant in terms of lymphopenia. There are reasons to believe the reliability of the predictive value of the hypothalamic dose. From the perspective of the maximal dose to the HT relating to lymphopenia in our results, these discrete nuclei may be interconnected functionally to participate in immunomodulatory activities. The speculation that the HT is a serial organ, at least with regard to immunoregulation, needs further verification.

These findings have important implications and provide insight into how the RT balance between antitumor efficacy and immunosuppression is relevant to clinical application. The effects of hypothalamic and brain dose on immunoregulation and survival underscore the need for dosimetric constraints during RT planning to mitigate functional complications. Modifying the dose that the HT and WB are exposed to may be feasible with improvements in RT planning with IMRT or proton therapy.^41^, ^42^

The strengths of our study are the uniform grouping criteria, and the limitation of the analysis is specifically related to the primary glioma patients treated with maximal surgical debulking and IMRT‐based therapy. Moreover, clinicopathological prognostic factors were included in the analysis and compared between lymphopenia subgroups to exclude confounders. We identified some notable shortcomings in previous studies, such as nonuniformity in the operative approach,^11^, ^14^, ^15^ diverse RT techniques,^14^ and relatively smaller populations,^9^, ^10^, ^11^, ^12^ that we believed might impede the reliability of the results. The major outstanding feature distinguished from previous studies was the enrollment of glioma patients with all WHO grades and the further subgroup analysis. LGG patients are generally long‐term survivors; potential late toxicities are of crucial importance and persistently affect survival quality, which should never be neglected. Concomitantly, considering the significant correlation between RT‐related lymphopenia and survival, we aimed to establish a direct link between dosimetric indicators and survival. This association held in the multivariate analysis after adjusting for other potential variables. Notably, this is the first report to demonstrate the prognostic value of dosimetric parameters on survival.

The principal limitations are recognized to be mainly associated with the retrospective nature of the study. Although insignificant differences in the distributions of basic clinicopathological factors were observed between the 2 groups, there could still be hidden confounding factors. Patients who were applied steroids were excluded, which affects the external validity of their results and conclusions. Moreover, reliable data on the phenotypes of lymphocytes and MGMT status were not available. As the overall importance, we will amend our protocol as these data are now available. In this regard, large‐scale prospective trials are needed to validate our results and confirm the clinical significance of these variables in this setting.

## 5 CONCLUSION

Treatment‐related lymphopenia is adverse to glioma patient survival. Higher doses to the hypothalamus (HT Dmax) or brain (WB Dmin, WB Dmean) are significantly correlated with the development of lymphopenia and a worse survival rate. Selective sparing of the HT and WB after exposure to doses below given thresholds may potentially provide a survival benefit after brain irradiation. The necessity and feasibility of dosimetric constraints for HT and WB is warranted with further investigation.

## CONFLICT OF INTEREST

The authors declare that they have no conflict of interest.
