# Supplementary figures and images for: Dosimetry of the brain and hypothalamus predicting acute lymphopenia and the survival of glioma patients with postoperative radiotherapy
Source: Cancer Med. 2019 Apr 14;8(6):2759–68. doi: 10.1002/cam4.2159 (PMC6558490; doi:10.1002/cam4.2159)

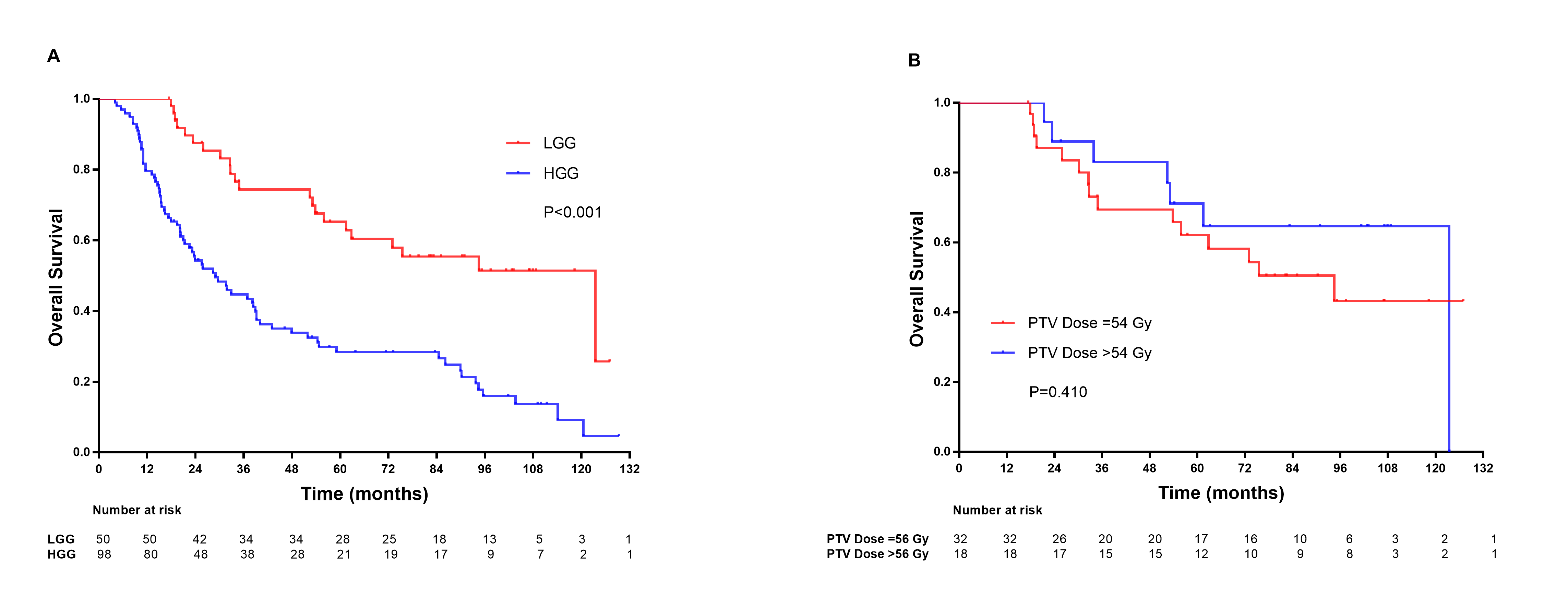

Supplement: Supplementary file 2 [file CAM4-8-2759-s002.tif]
