# Supplementary material for: Dosimetry of the brain and hypothalamus predicting acute lymphopenia and the survival of glioma patients with postoperative radiotherapy
Source: Cancer Med. 2019 Apr 14;8(6):2759–68. doi: 10.1002/cam4.2159 (PMC6558490; doi:10.1002/cam4.2159)
Supplement: Supplementary file 3 [file CAM4-8-2759-s003.doc]

**Supplementary Table 1. Baseline characteristics of 148 glioma patients treated with postoperative radiotherapy**

| Variables | Lowest lymphocyte count during radiotherapy (*10^9^/L) | | | |
| --- | --- | --- | --- | --- |
|  | Total (n=148) | Non-AL (n=79) | AL (n=69) | P value |
| *Age (years)* | 46.7±13.3 | 46.3±14.1 | 47.2±12.5 | 0.696^b^ |
| *Sex* |  |  |  | 0.068^a^ |
| Male | 93 (62.8) | 55 (69.6) | 38 (55.1) |  |
| Female | 55 (37.2) | 24 (30.4) | 31 (44.9) |  |
| *Site of tumor* |  |  |  | 0.097^a^ |
| Frontal lobe | 48 (32.4) | 33 (41.8) | 15 (21.7) |  |
| Temporal lobe | 47 (31.8) | 19 (24.1) | 28 (40.6) |  |
| Occipital lobe | 8 (5.4) | 4 (5.1) | 4 (5.8) |  |
| Parietal lobe | 8 (5.4) | 4 (5.1) | 4 (5.8) |  |
| Trans-lobal | 37 (25.0) | 19 (24.1) | 18 (26.1) |  |
| *Histological grade (WHO)* |  |  |  | 0.238^a^ |
| II | 50 (33.8) | 31 (39.2) | 19 (27.5) |  |
| III | 33 (22.3) | 18 (22.8) | 15 (21.7) |  |
| IV | 65 (43.9) | 30 (38.0) | 35 (50.7) |  |
| *PTV (cm^3^)* | 426.6±186.5 | 415.9±181.8 | 438.9±192.2 | 0.456^b^ |
| *Duration from surgery to radiation (weeks)* | 6.6±3.1 | 6.8±2.9 | 6.5±3.2 | 0.505^b^ |
| *Prescribed dose (Gy)* | 58.4±2.6 | 58.1±2.7 | 58.8±2.4 | 0.096^b^ |
| *Fraction* | 29±1 | 29±1 | 30±1 | 0.059^b^ |
| *Concomitant temozolomide* |  |  |  | **0.001**^a^ |
| No | 57 (38.5) | 40 (50.6) | 17 (24.6) |  |
| Yes | 91 (61.5) | 39 (49.4) | 52 (75.4) |  |
| *Hypothalamus DVH (Gy)* |  |  |  |  |
| Dmin | 32.9±16.5 | 29.9±17.4 | 35.7±15.0 | **0.031**^b^ |
| Dmax | 52.5±13.5 | 49.5±16.1 | 55.9±8.8 | **0.002**^b^ |
| Dmean | 43.4±15.7 | 40.5±17.6 | 46.7±12.4 | **0.015**^b^ |
| *Hippocampus DVH (Gy)* |  |  |  |  |
| Dmin | 17.0±11.6 | 15.9±12.6 | 18.3±10.3 | 0.206^b^ |
| Dmax | 55.7±12.6 | 54.1±13.5 | 57.5±11.4 | 0.104^b^ |
| Dmean | 36.8±13.9 | 34.3±14.9 | 39.5±12.2 | **0.022**^b^ |
| *Whole Brain DVH (Gy)* |  |  |  |  |
| Dmin | 2.4±1.9 | 2.0±1.4 | 2.9±2.3 | **0.006**^b^ |
| Dmax | 61.7±7.2 | 61.3±7.1 | 62.2±7.3 | 0.419^b^ |
| Dmean | 33.0±8.7 | 31.1±8.5 | 35.1±8.6 | **0.005**^b^ |

*Abbreviations:* cm^3^, cubic centimetre; Dmax, maximal dose; Dmean, mean dose; Dmin, minimal dose; DVH, dose-volume histogram; Gy, gray; PTV, planning target volume; WHO, World Health Organization.

^a^Chi-square test or Fischer’s exact test, P<0.05.

^b^Mann-Whitney U test, P<0.05.
